# 3-Chloro­benzohydrazide

**DOI:** 10.1107/S1600536810029508

**Published:** 2010-07-31

**Authors:** Uzma Ashiq, Rifat Ara Jamal, Muhammad Nadeem Arshad, Islam Ullah Khan

**Affiliations:** a Department of Chemistry, University of Karachi, Karachi 75270, Pakistan; b Department of Chemistry, Government College University, Lahore, Pakistan

## Abstract

In the title compound, C_7_H_7_ClN_2_O, the hydrazide group is inclined at a dihedral angle of 32.30 (11)° with respect to the benzene ring. The amino H atoms form inter­molecular N—H⋯O hydrogen bonds with the O atoms of two adjacent mol­ecules, resulting in 10-membered rings of graph-set motif *R*_2_^2^(10). The imino H atom is also involved in an inter­molecular hydrogen bond with an amino N atom of a symmetry-related mol­ecule, resulting in a zigzag chain along the *b* axis. The structure is further consolidated by an intra­molecular N—H⋯O inter­action, which results in a five-membered ring.

## Related literature

For the biological activity of hydrazides, see: Ashiq, Ara *et al.* (2008 ▶); Ara *et al.* (2007 ▶); Maqsood *et al.* (2006 ▶); For related structures, see: Ashiq, Jamal *et al.* (2008 ▶); Jamal *et al.* (2008 ▶, 2009 ▶); Kallel *et al.* (1992 ▶); Ratajczak *et al.* (2001 ▶); Saraogi *et al.* (2002 ▶). For graph-set notation of hydrogen-bond motifs, see: (Bernstein *et al.* 1995 ▶).
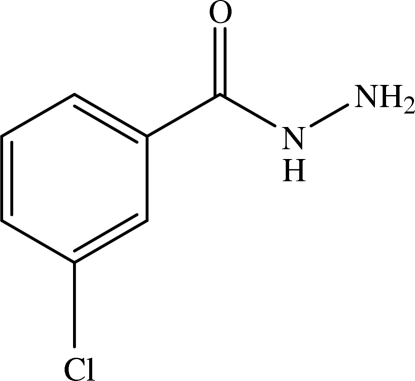


## Experimental

### 

#### Crystal data

C_7_H_7_ClN_2_O
*M* *_r_* = 170.60
Monoclinic, 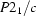

*a* = 16.2005 (15) Å
*b* = 3.8165 (4) Å
*c* = 12.7646 (13) Å
β = 108.030 (5)°
*V* = 750.47 (13) Å^3^
*Z* = 4
Mo *K*α radiation
μ = 0.45 mm^−1^
*T* = 296 K
0.43 × 0.21 × 0.17 mm

#### Data collection

Bruker Kappa APEXII CCD diffractometer
Absorption correction: multi-scan (*SADABS*; Bruker, 2005 ▶) *T*_min_ = 0.832, *T*_max_ = 0.928
8144 measured reflections
1881 independent reflections
1101 reflections with *I* > 2σ(*I*)
*R*_int_ = 0.053

#### Refinement

*R*[*F* ^2^ > 2σ(*F* ^2^)] = 0.049
*wR*(*F* ^2^) = 0.121
*S* = 1.01
1880 reflections
109 parameters
H atoms treated by a mixture of independent and constrained refinement
Δρ_max_ = 0.23 e Å^−3^
Δρ_min_ = −0.30 e Å^−3^

### 

Data collection: *APEX2* (Bruker, 2007 ▶); cell refinement: *SAINT* (Bruker, 2007 ▶); data reduction: *SAINT*; program(s) used to solve structure: *SHELXS97* (Sheldrick, 2008 ▶); program(s) used to refine structure: *SHELXL97* (Sheldrick, 2008 ▶); molecular graphics: *ORTEP-3 for Windows* (Farrugia, 1997 ▶); software used to prepare material for publication: *SHELXL97*.

## Supplementary Material

Crystal structure: contains datablocks I, global. DOI: 10.1107/S1600536810029508/pv2308sup1.cif

Structure factors: contains datablocks I. DOI: 10.1107/S1600536810029508/pv2308Isup2.hkl

Additional supplementary materials:  crystallographic information; 3D view; checkCIF report

## Figures and Tables

**Table 1:** Hydrogen-bond geometry (Å, °)

*D*—H⋯*A*	*D*—H	H⋯*A*	*D*⋯*A*	*D*—H⋯*A*
N2—H2*N*⋯O1	0.90 (3)	2.45 (3)	2.766 (3)	101 (2)
N1—H1*N*⋯N2^i^	0.87 (3)	2.11 (3)	2.955 (3)	162 (2)
N2—H3*N*⋯O1^ii^	0.86 (3)	2.24 (3)	3.091 (3)	171 (2)
N2—H2*N*⋯O1^iii^	0.90 (3)	2.25 (3)	2.935 (3)	133 (2)

Symmetry codes: (i) 
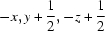
; (ii) 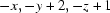; (iii) 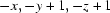.

**Fig. 1.**
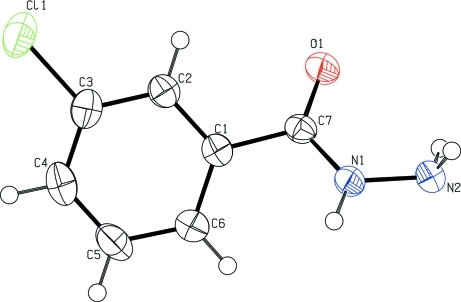
The molecular structure of the title compound with displacement ellipsoids drawn at 50% probability level.

**Fig. 2.**
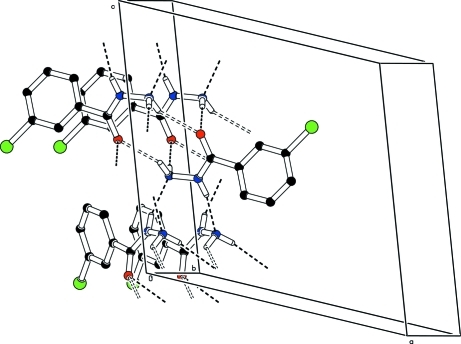
A packing diagram of the title compound; hydrogen bonds are shown by dashed lines.

## supplementary crystallographic information

### Comment

Hydrazides are known to have different biological activities (Ashiq, Ara *et al.*, 2008; Ara *et al.*, 2007). In order to study the biological activity of 3-chlorobenzohydrazide, we undertook the synthesis of the title compound and report its crystal structure in this paper. The title compound was found to be antifungal (Maqsood *et al.*, 2006). The structures of benzhydrazide (Kallel *et al.*, 1992), and its *p*-chloro (Saraogi *et al.*, 2002), *m*-methoxy (Jamal *et al.*, 2009), *m*-nitro (Ratajczak *et al.* 2001), *p*-bromo (Ashiq, Jamal *et al.*, 2008) and *p*-iodo (Jamal *et al.*, 2008) analogues have been reported.

The bond lengths and bond angles in the title compound (Fig. 1) are comparable with the corresponding distances and angles reported in its analogues quoted above. The hydrazide moiety, C7/O1/N1/N2, is oriented at a dihedral angle of 32.30 (11)° with respect to the plane of benzene ring C1–C6. The H atoms bonded to N2 form intermolecular hydrogen bonds of the type N–H···O with O atoms of two adjacent molecules, resulting in 10-membered rings which may be assigned to R_2_^2^(10) motif in graph set notation (Bernstein *et al.*, 1995). The H-atom bonded to N1 is also involved in an intermolecular hydrogen bond with N2, linking the molecules into a zigzag chain along the *b* axis (Tab. 1 and Fig. 2). The structure is further stabilized by an intramolecular interaction, N2–H2···O1, resulting in a five membered ring in S(5) motif (Bernstein *et al.*, 1995).

### Experimental

All reagent-grade chemicals were obtained from Aldrich and Sigma Chemical companies and were used without further purification. To a solution of ethyl-3-chlorobenzoate (3.69 g, 20 mmol) in 75 ml ethanol, hydrazine hydrate (5.0 ml, 100 mmol) was added. The mixture was refluxed for 5 h and a solid was obtained upon removal of the solvent by rotary evaporation. The resulting solid was washed with hexane to afford the title compound (yield 75%). The crystals of the title compound suitable for crystallographic study were grown from a solution of methanol by slow evaporation at room temperature.

### Refinement

H atoms were positioned geometrically, with C—H = 0.93 Å for aromatic and constrained to ride on their parent atoms. The H-atoms attached to N1 and N2 were taken from Fourier maps and their coordinates were refined. The thermal parameters, *U*_iso_, of H-atoms were allowed at 1.2 times of the *U*_eq_ of their parent atoms.

### Crystal data

**Table:**

C_7_H_7_ClN_2_O	*F*(000) = 352
*M**_r_* = 170.60	*D*_x_ = 1.510 Mg m^−^^3^
Monoclinic, *P*2_1_/*c*	Mo *K*α radiation, λ = 0.71073 Å
Hall symbol: -P 2ybc	Cell parameters from 1323 reflections
*a* = 16.2005 (15) Å	θ = 3.2–23.3°
*b* = 3.8165 (4) Å	µ = 0.45 mm^−^^1^
*c* = 12.7646 (13) Å	*T* = 296 K
β = 108.030 (5)°	Needle, colorless
*V* = 750.47 (13) Å^3^	0.43 × 0.21 × 0.17 mm
*Z* = 4	

### Data collection

**Table:**

Bruker Kappa APEXII CCD diffractometer	1881 independent reflections
Radiation source: fine-focus sealed tube	1101 reflections with *I* > 2σ(*I*)
graphite	*R*_int_ = 0.053
ω scans	θ_max_ = 28.5°, θ_min_ = 1.3°
Absorption correction: multi-scan (*SADABS*; Bruker, 2005)	*h* = −21→21
*T*_min_ = 0.832, *T*_max_ = 0.928	*k* = −5→5
8144 measured reflections	*l* = −17→17

### Refinement

**Table:**

Refinement on *F*^2^	Primary atom site location: structure-invariant direct methods
Least-squares matrix: full	Secondary atom site location: difference Fourier map
*R*[*F*^2^ > 2σ(*F*^2^)] = 0.049	Hydrogen site location: inferred from neighbouring sites
*wR*(*F*^2^) = 0.121	H atoms treated by a mixture of independent and constrained refinement
*S* = 1.01	*w* = 1/[σ^2^(*F*_o_^2^) + (0.0424*P*)^2^ + 0.4337*P*] where *P* = (*F*_o_^2^ + 2*F*_c_^2^)/3
1880 reflections	(Δ/σ)_max_ < 0.001
109 parameters	Δρ_max_ = 0.23 e Å^−^^3^
0 restraints	Δρ_min_ = −0.30 e Å^−^^3^

### Special details

**Table:**

Experimental. the reflection 1 0 0 has been obscured by the beam stop so it was omitted in the final refinement
Geometry. All s.u.'s (except the s.u. in the dihedral angle between two l.s. planes) are estimated using the full covariance matrix. The cell s.u.'s are taken into account individually in the estimation of s.u.'s in distances, angles and torsion angles; correlations between s.u.'s in cell parameters are only used when they are defined by crystal symmetry. An approximate (isotropic) treatment of cell s.u.'s is used for estimating s.u.'s involving l.s. planes.
Refinement. Refinement of *F*^2^ against ALL reflections. The weighted *R*-factor *wR* and goodness of fit *S* are based on *F*^2^, conventional *R*-factors *R* are based on *F*, with *F* set to zero for negative *F*^2^. The threshold expression of *F*^2^ > 2σ(*F*^2^) is used only for calculating *R*-factors(gt) *etc*. and is not relevant to the choice of reflections for refinement. *R*-factors based on *F*^2^ are statistically about twice as large as those based on *F*, and *R*- factors based on ALL data will be even larger.

### Fractional atomic coordinates and isotropic or equivalent isotropic displacement parameters (Å^2^)

**Table:**

	*x*	*y*	*z*	*U*_iso_*/*U*_eq_	
Cl1	0.43417 (4)	1.3067 (2)	0.63671 (7)	0.0544 (3)	
O1	0.11735 (11)	0.7193 (5)	0.53677 (14)	0.0392 (5)	
N1	0.05596 (12)	0.8834 (6)	0.36054 (17)	0.0336 (5)	
H1N	0.0600 (15)	0.987 (8)	0.301 (2)	0.040*	
N2	−0.02733 (13)	0.7486 (7)	0.35190 (18)	0.0352 (6)	
H3N	−0.0560 (16)	0.902 (8)	0.376 (2)	0.042*	
H2N	−0.0219 (16)	0.571 (8)	0.399 (2)	0.042*	
C1	0.20977 (14)	0.9403 (7)	0.43785 (19)	0.0295 (6)	
C2	0.27380 (15)	1.0618 (7)	0.5299 (2)	0.0331 (6)	
H2	0.2629	1.0822	0.5971	0.040*	
C3	0.35388 (15)	1.1523 (7)	0.5212 (2)	0.0365 (6)	
C4	0.37114 (17)	1.1191 (8)	0.4231 (2)	0.0445 (7)	
H4	0.4252	1.1827	0.4180	0.053*	
C5	0.30846 (17)	0.9917 (9)	0.3325 (2)	0.0472 (8)	
H5	0.3205	0.9645	0.2663	0.057*	
C6	0.22744 (16)	0.9033 (8)	0.3388 (2)	0.0380 (7)	
H6	0.1849	0.8194	0.2768	0.046*	
C7	0.12403 (15)	0.8398 (7)	0.45054 (19)	0.0286 (5)	

### Atomic displacement parameters (Å^2^)

**Table:**

	*U*^11^	*U*^22^	*U*^33^	*U*^12^	*U*^13^	*U*^23^
Cl1	0.0334 (3)	0.0599 (5)	0.0633 (5)	−0.0054 (3)	0.0055 (3)	−0.0109 (4)
O1	0.0392 (9)	0.0492 (13)	0.0309 (9)	−0.0036 (9)	0.0133 (7)	0.0086 (9)
N1	0.0321 (11)	0.0414 (15)	0.0281 (11)	−0.0062 (10)	0.0106 (9)	0.0069 (10)
N2	0.0312 (11)	0.0440 (17)	0.0331 (12)	−0.0057 (10)	0.0138 (9)	−0.0015 (11)
C1	0.0310 (12)	0.0254 (14)	0.0336 (13)	0.0014 (10)	0.0120 (10)	0.0031 (11)
C2	0.0338 (12)	0.0323 (16)	0.0351 (14)	−0.0003 (11)	0.0131 (11)	0.0021 (12)
C3	0.0305 (12)	0.0293 (16)	0.0469 (16)	0.0012 (11)	0.0081 (11)	0.0013 (13)
C4	0.0319 (13)	0.0456 (19)	0.0609 (19)	0.0030 (13)	0.0216 (13)	0.0055 (15)
C5	0.0458 (16)	0.058 (2)	0.0472 (17)	0.0028 (15)	0.0282 (13)	0.0043 (16)
C6	0.0369 (13)	0.0451 (19)	0.0340 (14)	−0.0006 (13)	0.0138 (11)	−0.0019 (13)
C7	0.0342 (12)	0.0253 (14)	0.0288 (12)	−0.0008 (11)	0.0132 (10)	−0.0018 (11)

### Geometric parameters (Å, °)

**Table:**

Cl1—C3	1.740 (3)	C1—C7	1.497 (3)
O1—C7	1.228 (3)	C2—C3	1.380 (3)
N1—C7	1.334 (3)	C2—H2	0.9300
N1—N2	1.416 (3)	C3—C4	1.371 (4)
N1—H1N	0.87 (3)	C4—C5	1.370 (4)
N2—H3N	0.86 (3)	C4—H4	0.9300
N2—H2N	0.90 (3)	C5—C6	1.381 (3)
C1—C2	1.385 (3)	C5—H5	0.9300
C1—C6	1.387 (3)	C6—H6	0.9300
			
C7—N1—N2	122.4 (2)	C2—C3—Cl1	119.4 (2)
C7—N1—H1N	122.6 (16)	C3—C4—C5	119.7 (2)
N2—N1—H1N	114.9 (16)	C3—C4—H4	120.1
N1—N2—H3N	109.4 (19)	C5—C4—H4	120.1
N1—N2—H2N	109.3 (16)	C4—C5—C6	120.5 (3)
H3N—N2—H2N	103 (3)	C4—C5—H5	119.8
C2—C1—C6	119.7 (2)	C6—C5—H5	119.8
C2—C1—C7	118.0 (2)	C5—C6—C1	119.7 (2)
C6—C1—C7	122.2 (2)	C5—C6—H6	120.1
C3—C2—C1	119.4 (2)	C1—C6—H6	120.1
C3—C2—H2	120.3	O1—C7—N1	122.7 (2)
C1—C2—H2	120.3	O1—C7—C1	122.3 (2)
C4—C3—C2	120.9 (2)	N1—C7—C1	115.0 (2)
C4—C3—Cl1	119.7 (2)		
			
C6—C1—C2—C3	1.5 (4)	C2—C1—C6—C5	−0.7 (4)
C7—C1—C2—C3	179.2 (2)	C7—C1—C6—C5	−178.3 (3)
C1—C2—C3—C4	−0.9 (4)	N2—N1—C7—O1	−9.3 (4)
C1—C2—C3—Cl1	179.7 (2)	N2—N1—C7—C1	169.2 (2)
C2—C3—C4—C5	−0.5 (4)	C2—C1—C7—O1	−31.5 (4)
Cl1—C3—C4—C5	178.9 (2)	C6—C1—C7—O1	146.2 (3)
C3—C4—C5—C6	1.3 (5)	C2—C1—C7—N1	150.0 (2)
C4—C5—C6—C1	−0.7 (5)	C6—C1—C7—N1	−32.3 (4)

### Hydrogen-bond geometry (Å, °)

**Table:**

*D*—H···*A*	*D*—H	H···*A*	*D*···*A*	*D*—H···*A*
N2—H2N···O1	0.90 (3)	2.45 (3)	2.766 (3)	101 (2)
N1—H1N···N2^i^	0.87 (3)	2.11 (3)	2.955 (3)	162 (2)
N2—H3N···O1^ii^	0.86 (3)	2.24 (3)	3.091 (3)	171 (2)
N2—H2N···O1^iii^	0.90 (3)	2.25 (3)	2.935 (3)	133 (2)

Symmetry codes: (i) −*x*, *y*+1/2, −*z*+1/2; (ii) −*x*, −*y*+2, −*z*+1; (iii) −*x*, −*y*+1, −*z*+1.
